# Current practice in methodology and reporting of the sample size calculation in randomised trials of hip and knee osteoarthritis: a protocol for a systematic review

**DOI:** 10.1186/s13063-017-2209-8

**Published:** 2017-10-10

**Authors:** Bethan Copsey, Susan Dutton, Ray Fitzpatrick, Sarah E. Lamb, Jonathan A. Cook

**Affiliations:** 1 Centre for Statistics in Medicine, Nuffield Department of Orthopaedics, Rheumatology and Musculoskeletal Sciences, University of Oxford, Oxford, UK; 2 Nuffield Department of Population Health, University of Oxford, Oxford, UK

**Keywords:** Sample size, Osteoarthritis, Reporting, Systematic review

## Abstract

**Background:**

A key aspect of the design of randomised controlled trials (RCTs) is determining the sample size. It is important that the trial sample size is appropriately calculated. The required sample size will differ by clinical area, for instance, due to the prevalence of the condition and the choice of primary outcome. Additionally, it will depend upon the choice of target difference assumed in the calculation. Focussing upon the hip and knee osteoarthritis population, this study aims to systematically review how the trial size was determined for trials of osteoarthritis, on what basis, and how well these aspects are reported.

**Methods:**

Several electronic databases (Medline, Cochrane library, CINAHL, EMBASE, PsycINFO, PEDro and AMED) will be searched to identify articles on RCTs of hip and knee osteoarthritis published in 2016. Articles will be screened for eligibility and data extracted independently by two reviewers. Data will be extracted on study characteristics (design, population, intervention and control treatments), primary outcome, chosen sample size and justification, parameters used to calculate the sample size (including treatment effect in control arm, level of variability in primary outcome, loss to follow-up rates). Data will be summarised across the studies using appropriate summary statistics (e.g. *n* and %, median and interquartile range). The proportion of studies which report each key component of the sample size calculation will be presented. The reproducibility of the sample size calculation will be tested.

**Discussion:**

The findings of this systematic review will summarise the current practice for sample size calculation in trials of hip and knee osteoarthritis. It will also provide evidence on the completeness of the reporting of the sample size calculation, reproducibility of the chosen sample size and the basis for the values used in the calculation.

**Trial registration:**

As this review was not eligible to be registered on PROSPERO, the summary information was uploaded to Figshare to make it publicly accessible in order to avoid unnecessary duplication amongst other benefits (https://doi.org/10.6084/m9.figshare.5009027.v1); Registered January 17, 2017.

**Electronic supplementary material:**

The online version of this article (doi:10.1186/s13063-017-2209-8) contains supplementary material, which is available to authorized users.

## Background

The sample size and target difference of a clinical trial is a key feature that impacts on how the trial is designed and conducted [1]. There are multiple factors which contribute to the determination of the required sample size with choice of target difference arguably the most important [2, 3]. It is recognised that an overly large sample size is undesirable as it increases the costs of the trial and likely delays dissemination of the findings [2]. Too large a sample size is also unethical as it may result in additional participants receiving a treatment when there is already sufficient evidence to show that it is inferior to an alternative [4]. Conversely, too small a sample size also poses ethical issues as it will result in a study lacking sufficient power to detect a clinically important treatment effect [5, 6].

Many studies have found that sample sizes are often inadequately reported and based on inaccurate assumptions [7, 8]. Discrepancies in the assumptions for parameters in sample size calculations can impact on power [9, 10]. In particular, for continuous outcomes, underestimation of the standard deviation can lead to underpowered studies. Tavernier and Giraudeau hypothesised that this mis-specification may be due to the imprecision and more homogeneous populations of pilot studies often used to estimate the parameter [9].

OARSI (Osteoarthritis Research Society International) recently published recommendations to use realistic and clinically important effect sizes when calculating the sample size for an osteoarthritis trial, suggesting that previously some trials have not done so [11]. Keen et al. found that rheumatology trials published in 2001–2002 were often underpowered and sample size calculations were poorly reported [12]. Since then, there have been considerable efforts to improve reporting of randomised trials; for example, the Consolidated Standards of Reporting Trials (CONSORT) Statement includes an item to report how the ‘sample size was determined’ [13]. However, it is unclear how investigators currently determine and report the sample size for trials of hip and knee osteoarthritis.

### Aim and objectives

The primary objective is to summarise the methodology used (including the assumptions made and justifications provided) to determine the sample size calculation for randomised trials of hip and knee osteoarthritis.

The secondary objectives are to assess the reporting and reproducibility of the sample size calculation.

## Methods

The PRISMA-P Checklist for this review protocol is available as Additional file 1.

### Inclusion criteria

Studies will be eligible for inclusion if they are randomised controlled trials of hip and/or knee osteoarthritis with two treatment arms (one intervention and one comparator) published in 2016. Inclusion will not be restricted by study outcomes, intervention or control treatments.

### Exclusion criteria

Articles on non-randomised studies will be excluded, including case-control or cross-sectional studies. Quasi-randomised studies or studies which do not state that the allocation was randomised will be excluded. Factorial design and cross-over trials will be excluded. Trials with more than two arms will be excluded.

Pilot studies will be excluded, as will studies which refer to themselves as ‘feasibility’, ‘proof of concept’ or ‘exploratory’ studies. Studies which intend to use results to inform future definitive phase III trials will not be included. Studies which do not consider treatment evaluation (e.g. compare different screening methods) will be excluded.

Studies on the prevention of osteoarthritis or trials with mixed populations will be excluded; for example, a combination of rheumatoid arthritis and osteoarthritis. Trials of, for example, total knee arthroplasty will only be eligible if it is clearly stated that all participants had knee osteoarthritis.

Non-English language articles will be excluded as this review focusses on study reporting.

Articles will be excluded if they are conference abstracts or study protocols. Only the primary report of a trial will be eligible; separate publications for secondary analyses will be excluded, including long-term follow-up or subgroup analyses.

### Identification of studies

Articles reporting the results of clinical trials will be identified using electronic searches of databases including Medline, Cochrane Central Register of Controlled Trials (CENTRAL), CINAHL, EMBASE, PsycINFO, PEDro and AMED. An example search strategy is given in Additional file *2*.

A preliminary search to inform the search strategy indicated that this would lead to around 100 included studies, which was considered to be sufficiently large for a methodological review of this kind [14–16].

### Selection of studies

All search results will be combined and duplicates will be removed. Titles and abstracts will be screened independently by two reviewers. Following this, full texts for the records considered to be potentially included will be obtained and also screened independently by two researchers. Any disagreements will be resolved through discussion between the two reviewers assessing the papers, with involvement of a further reviewer where necessary.

### Data extraction and management

Data will be extracted using a standardised form. The data-extraction form will be piloted to ensure that all relevant information is recorded and to allow refinement prior to formal use. The form will then be enhanced by adding and clarifying items to extract. Data extraction will be performed by a second reviewer on a sample of included studies (at least 20%) in order to check accuracy. Relevant data will be extracted from the study protocol where this is cited in the main trial results publication; where there are conflicts between the information in the protocol and main publication, information from the main publication will be used.

The following information will be extracted from each article when reported:

- Article: Country
- Design: Study design (e.g. superiority, non-inferiority)
- Population: Condition (including how osteoarthritis was defined), setting, eligibility criteria (in particular age, gender, and disease severity).
- Treatment: Intervention, comparator.
- Outcome: Primary outcome measure(s)
- Sample size details: Statistical approach (conventional, other), chosen sample size, method for calculation, values used and justification (e.g. effect size, target difference, standard deviation, adjustment for loss to follow-up, sidedness, significance level, power), whether sample size could be replicated, whether sample size re-estimation was planned (e.g. using interim analysis), whether sensitivity analysis was conducted to examine impact of assumptions on sample size. Note: Post-hoc sample size calculations will not be considered.
- Follow-up: Number of participants randomised, number lost to follow-up, whether compliance was measured.

### Data synthesis

Data will be summarised across the studies, including the general characteristics of the included studies using appropriate summary statistics (e.g. *n* and %, median and interquartile range (IQR)).

The proportion of studies that justify the sample size and target difference, and which report each component of the sample size calculation will be calculated.

The target difference expressed as a standardised effect size (e.g. Cohen’s *d* for continuous outcome) will be presented graphically to compare across the studies and, where there are a sufficient number of studies, within conditions (i.e. considering hip osteoarthritis and knee osteoarthritis separately) [17].

### Sample size replication

Using the reported values, an attempt will be made to replicate the sample size calculation. It will be assumed that 80% power and 5% two-sided significance level were used unless otherwise stated and that a conventional (Neyman-Pearson) approach to the sample size calculation has been adopted [1]. The sample size calculation will be replicated using statistical software such as the ‘power twomeans’ command in STATA IC 14 [18].

When comparing the replicated to stated sample size, the ratio of replicated/reported values will be calculated. Ratios will be presented in a box plot [19]. The proportion of studies where the ratio is ≥ 1.1 or ≤ 0.9, and ≥ 1.3 or ≤ 0.7 (i.e. out by at least 10% or 30%) will be calculated.

### Subgroup analysis

Subgroup analysis will be used to explore the associations between study-level characteristics and key aspects of the sample size calculation: (1) observed sample size (number of participants randomised), (2) whether the sample size calculation was fully specified and (3) replicability. Data will be summarised within subgroups and presented using box plots (median, interquartile range (IQR) and range).

For subgroup comparisons, the following aspects will be compared:

1. Type of intervention: surgical vs non-surgical trials
2. Centres: single vs multi-centre
3. Funding: industry-funded (all or in part) vs no industry funding (or not reported)
4. Comparator: placebo/waitlist vs active control

If sufficient studies are reflected across the respective subgroups, we will formally compare groups: (1) sample size will be compared between subgroups using the median difference and 95% confidence interval. Absolute risk differences with 95% confidence intervals will be used to compare between subgroups for (2) reporting of a sample size calculation, (3) reporting of core sample size components and (4) the replicated sample size being > 10% larger than the reported sample size.

Formal subgroup comparisons will only be conducted where a sufficient number of studies are present within each group. A two-sided significance level of 0.05 will be used. As the subgroup analysis is exploratory, no adjustment will be made for multiple testing.

## Discussion

This review will examine the current practice for sample size calculation in randomised trials of hip and knee osteoarthritis, which will include the target difference that studies are designed to detect, the chosen sample size and justification of key inputs. It will also provide evidence on the completeness of the reporting of the sample size calculation and the accuracy of the sample size calculation (i.e. whether the calculation was reproducible). This systematic review will also provide insight into the number of randomised trials being conducted on hip and knee osteoarthritis and the variety of interventions being evaluated. Focussing on a specific clinical area will permit a more detailed assessment of the methodology within a more homogeneous sample of trials.

Subgroup analysis will explore whether the sample size used and reporting of the sample size calculation differ based on type of intervention, number of centres, funding source and comparator. Surgical and non-surgical studies will be compared since several studies have highlighted the complexities of surgical trials and have highlighted their poor methodological quality and reporting [20–22]. Studies have also suggested that multi-centre trials may have higher methodological quality than single-centre trials [20, 23, 24]. The effect of funding source will be examined since previous reviews have shown that industry-funded studies may differ in terms of transparency and outcome reporting [25–27]. Finally, trials with an active comparator will be compared to those with a placebo or ‘no treatment’ control since trials with an active control arm may have methodological differences, e.g. using a smaller target difference and thus requiring a larger sample size [28, 29].

This systematic review will be limited in that it relies primarily on information from the trials’ results publication(s), which may not be transparent about modification once the study had begun and thus may not accurately reflect the a-priori sample size calculation when the study was planned. There is some evidence to suggest that practice is more complex than trial reports suggest [30]. Nevertheless, the reported sample size should reflect the final design and is the natural one to assess, at least in the first instance.

The findings of this systematic review will provide evidence on whether sufficient information is being reporting in sample size calculations and explore variability in the chosen sample size and reporting based on study design features (including the justification of key inputs). This may highlight areas for improvement in the reporting and conduct of sample size calculations for hip and knee osteoarthritis trials, and to an extent, trials of other conditions.

## Additional files

Additional file 1: This includes the PRISMA Checklist. The line numbers relate to the original manuscript submission. (DOCX 29 kb)

Additional file 2: This details the search terms used for the MEDLine database. (DOCX 13 kb)

## Abbreviations

RCT: Randomised controlled trial
IQR: Interquartile range
OARSI: Osteoarthritis Research Society International
