# Supplementary material for: Current practice in methodology and reporting of the sample size calculation in randomised trials of hip and knee osteoarthritis: a protocol for a systematic review
Source: Trials. 2017 Oct 10;18:466. doi: 10.1186/s13063-017-2209-8 (PMC5634891; doi:10.1186/s13063-017-2209-8)
Supplement: Supplementary file 2 — This details the search terms used for the MEDLine database. (DOCX 13 kb) [file 13063_2017_2209_MOESM2_ESM.docx]

**Appendix 2. Search Strategy Example (MEDLINE)**

1 randomized controlled trial.pt.

2 controlled clinical trial.pt.

3 randomized.ab.

4 placebo.ab.

5 clinical trial/

6 randomly.ab.

7 trial.ti.

8 1 or 2 or 3 or 4 or 5 or 6 or 7

9 humans/

10 8 and 9

11 exp Osteoarthritis/

12 osteoarthr$.tw.

13 (degenerative adj3 (arthr$ or joint$ or disease$)).tw.

14 arthros?s.tw.

15 11 or 12 or 13 or 14

16 10 and 15
